# Why should we assess the degree of frailty in primary care?

**DOI:** 10.3399/BJGP.2025.0772

**Published:** 2026-06-25

**Authors:** Peter Hanlon, Deb Gompertz, Holly Paris, Kenneth Rockwood

**Affiliations:** 1 General Practice and Primary Care, School of Health and Wellbeing, University of Glasgow, Glasgow, UK; 2 Rural Integrated Neighbourhood Team, Somerset Partnership NHS Foundation Trust, Somerset, UK; 3 Pier Health Group, Weston-super-Mare, UK; 4 Department of Geriatric Medicine, Dalhousie University, Halifax, Canada; 5 Frailty and Elder Care Network, Nova Scotia Health, Halifax, Canada

Caring for people living with frailty is core business for general practice. When identifying frailty, clinicians often say they ‘know it when they see it’.^1^ Increasingly, primary care is encouraged to assess frailty more explicitly: to name it, score it, and code it. Many question what value this adds.^1,2^ Here, we outline why we believe identifying and communicating the degree of frailty is a key aspect in delivering personalised care to older people within primary care.

## What is frailty?

Frailty describes a person’s vulnerability to adverse health outcomes. More specifically, it expresses reduced ability to resist potentially damaging stress (‘robustness’), such as an infection, a drug side effect, or a fall, coupled with delayed or incomplete recovery following a physiological insult (‘resilience’).^3^ Practically, these events manifest as delirium, falls, reduced mobility, social withdrawal, and loss of independence. Often acting together, these factors increase the need for care.^4^ These events often feel sudden and unexpected to patients and relatives. However, frailty assessment allows us to judge the likelihood of these acute events and, with the benefit of continuity, understand a patient’s trajectory. A proactive approach to frailty, informed by an accurate assessment of frailty severity, allows these events to be anticipated, planned for, or prevented, and for future care needs to be planned for and delivered in ways that align with patients’ values and priorities.^5^

## Why assess frailty severity?

**Figure 1.**
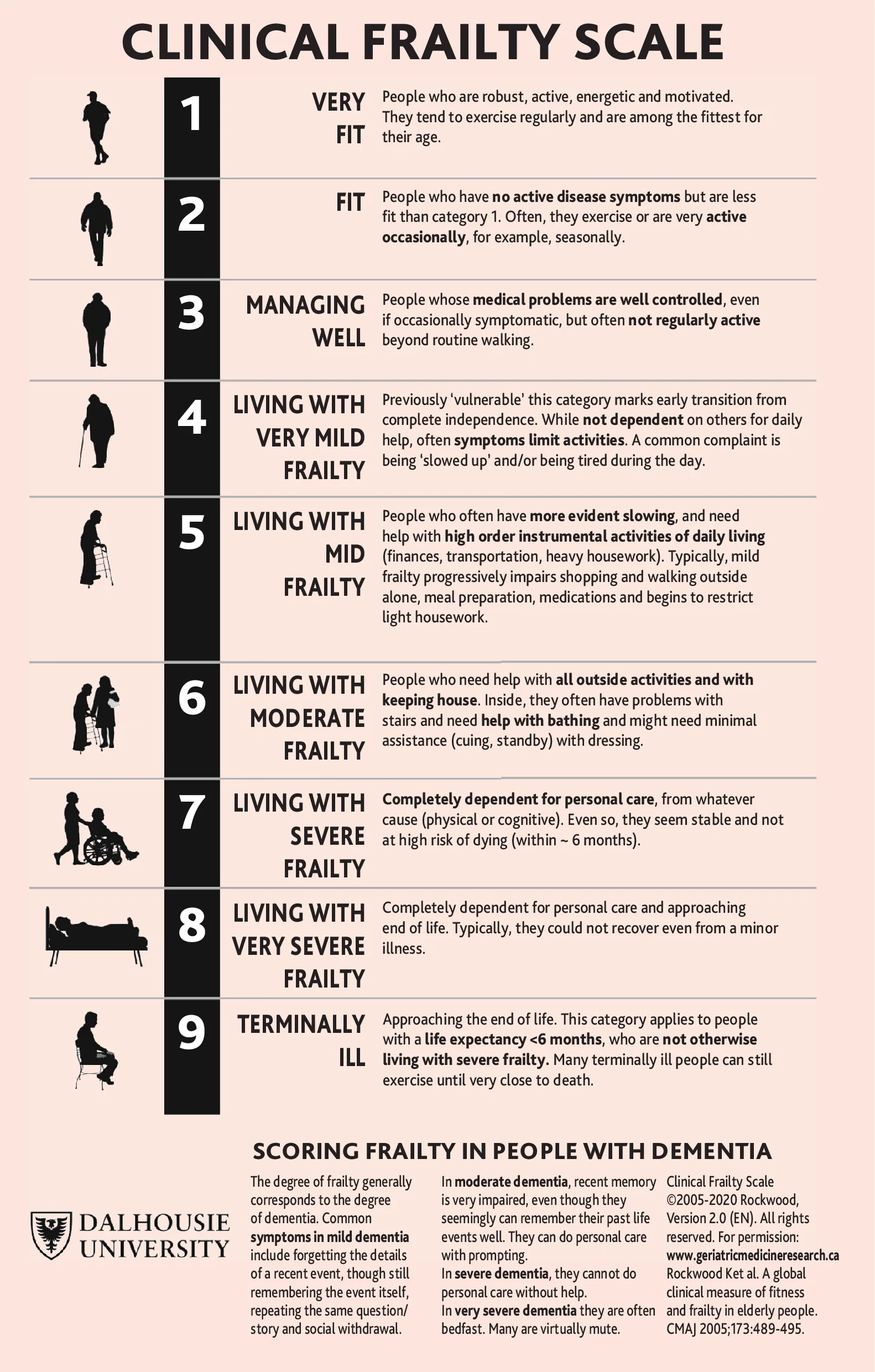
The Clinical Frailty Scale. Image reproduced with permission (Dalhousie University). More detail on the scale, including a decision tree for patient classification and information on permissions for reuse, can be found at https://www.dal.ca/sites/gmr/our-tools/clinical-frailty-scale.html.

Frailty is not all or nothing: it exists by degree. The prognosis, trajectory, and care needs of someone with mild frailty are vastly different from someone living with severe frailty. Being specific and accurate about an individual’s frailty status can facilitate care that reflects this. In addition, identifying frailty at the population level can aid more effective allocation and commissioning of services, reflecting the fact that people with more severe frailty have both an increased requirement for health and social care services in general, and a greater need for services that are attuned to their specific needs. Passive measures such as the Electronic Frailty Index, now in its second iteration, can be helpful in stratifying practice populations to identify people at greatest risk of frailty.^6^ However, to identify frailty at an individual level, different tools are required. A good option is the Clinical Frailty Scale, a 9-point scale reflecting high-order age-related deficits that tend to accumulate as people age (Figure 1).^7^ This tool allows an individual-level assessment of frailty, informed by their level of functional independence. From this can come a more precise and accurate assessment of an individual’s degree of frailty. It does, however, require clinical assessment. Access to primary care and proactive approaches to frailty identification are therefore central to effective frailty assessment. This is particularly important given the inextricable links between frailty, socioeconomic deprivation, and social vulnerability.^8,9^

## What can we do about frailty?

Identifying frailty only adds value if it affects what we do. Grading the degree of frailty opens up opportunities to deliver care that is frailty attuned. There are a number of practical steps that are possible:

### Pragmatic prescribing

People living with frailty deserve high-quality, evidence-based treatment. However, people with severe frailty are rarely included in randomised controlled trials that inform clinical guidelines. As a result, as the degree of frailty increases, the applicability of standard disease-specific recommendations becomes more questionable. Dealing with this uncertainty demands a pragmatic approach to balancing risk and benefit. Defining a patient’s degree of frailty can enable this to happen.

In November 2025, the British Geriatrics Society launched a one-page guideline on pragmatic prescribing for moderate to severe frailty.^10^ The guidance highlights common scenarios where the risks of standard, disease-specific treatments can begin to exceed the benefits for people with moderate or severe frailty. Importantly, recommendations differ depending on the degree of frailty, and emphasise flexibility, sharing of options, and individualised decision making. Shared decision making is a core skill in the general practice toolkit. Frailty assessment accompanied by clear guidance such as this can therefore facilitate shared treatment decisions that are backed by clear guidance.

This guidance is endorsed by the Royal College of General Practitioners and the Royal College of Physicians, and the National Institute for Health and Care Excellence has confirmed that this pragmatic approach is supported by wider guidance including NG56 — *Multimorbidity: Clinical Assessment and Management*. Support has not been universal, however, and some (such as the British Society for Heart Failure) have expressed concern that the guidance goes against evidence-based practice supported by randomised controlled trial evidence. While such views can appear contradictory, they are largely driven by a focus on different parts of the frailty spectrum.^11^ Randomised controlled trial participants, even in trials focusing on older people, typically have much less severe degrees of frailty than those living with moderate or severe frailty for whom pragmatic prescribing is advocated. Where trials have been conducted in severely frail populations, findings often support a more pragmatic approach. For example, in the recent RETREAT-FRAIL trial of deprescribing in hypertension, they demonstrate that deintensification and higher blood pressure targets are safe in people living with frailty.^12^ This also demonstrates that, while difficult, trials including people with moderate and severe frailty are possible. Accumulating more such evidence and testing the impact of pragmatic prescribing across indications and settings, will be important to ensure older people living with frailty can benefit from the highest standards of evidence, designed with their specific needs in mind.

### Targeted intervention

Evidence-based interventions for frailty exist, and identifying people living with frailty can facilitate access to these interventions. Comprehensive Geriatric Assessment (CGA) is a multidisciplinary intervention involving holistic assessment with care planning and delivery that seeks to address the broad needs of older people.^5^ CGA has potential to reduce unplanned hospital admissions,^13^ including when delivered within primary care,^14^ although evidence for impact on other outcomes such as mortality, functional independence, or quality of life is less clear. As models of primary care develop and evolve, with greater emphasis on proactive care for frailty, CGA is increasingly likely to inform the underlying principles of multidisciplinary teams targeting frailty within primary care. Knowing what works, for whom, and in what circumstances is going to be increasingly important, as is understanding what components of complex interventions are key to improving outcomes for older people living with frailty.

A landmark network meta-analysis published in 2024 synthesised evidence of community-based complex interventions to sustain independence for older people. Crucially, they analysed what components of interventions were most associated with improvement in independence. The analysis demonstrated that individualised care planning with medicines optimisation and regular follow-up review was most effective in sustaining independence among older people, with those receiving care at home particularly likely to benefit.^15^ Certainty was lower for other combinations and, somewhat surprisingly, some combinations of components appeared to reduce functional independence among older people. Few of the included studies explicitly assessed frailty. This highlights several crucial points: improving functional independence for older people is possible, but we cannot assume that all efforts to achieve this will be effective and some may have the opposite effect. Those most likely to be effective also involved sustained follow-up. As primary care teams develop innovative approaches to managing frailty, this need for careful consideration of what is being offered, the need for sustained engagement, and the potential for harm, are key considerations.

Evidence for narrower interventions to improve frailty is emerging, including from several trials conducted in primary care.^16^ Strength training and nutritional optimisation hold particular promise. While work is ongoing to define precisely for whom, in what ‘dose’, and at what time these interventions are most effective, this emerging evidence base emphasises that frailty identification can highlight opportunities to intervene to help improve or optimise a patient’s level of function. GPs and primary care teams are well versed in promoting physical activity, dietary interventions, and supporting patients to make changes. Frailty should not be a barrier to this, and indeed people living with frailty may have a lot to gain.

Access to these interventions will depend on local provision and availability. However, an awareness of their potential, and an understanding that frailty does not indicate futility in attempting to improve function or independence, is an essential starting point. Primary care teams are evolving, as are models of complex care delivery. Where teams exist that can deliver CGA, identifying people with advancing frailty can help select those most likely to benefit. As social prescribing and multidisciplinary primary care teams become more widespread, this has potential to provide opportunities for tailored intervention. Promoting exercise or social engagement should be a key part of what we can deliver. We should emphasise, however, that the evidence for interventions such as social prescribing and CGA — particularly delivered in primary care — is continuing to develop. As initiatives such as primary care link workers become more widespread, this should be accompanied by careful and rigorous evaluation. Importantly, the evidence base for social prescribing has largely lacked explicit consideration of frailty. Understanding if and how people with frailty access such services, how they are best deployed to meet the needs of people living with frailty, and assessing what impact this has on outcomes prioritised by people living with frailty and their caregivers, should be priorities for primary care research. Partnership between researchers, healthcare services, third sector and community organisations is likely to be key to achieving these goals.

### Anticipating needs and trajectories

Identifying someone’s degree of frailty can inform assessment of their current and future care needs and allow informed judgements to be made about their likely response to illness.

An individual with mild frailty can experience slowing down, accompanied by some difficulty with more complex tasks. Often, they can maintain independence and can expect to live a long time with some degree of frailty. Identification of mild frailty can be a trigger to anticipate when additional support may be required, or that vulnerability to unexpected decline in health may be lurking.

In contrast, moderate to severe frailty is associated with a 3-year mortality of around 50%.^17^ Ninety-day survival following emergency department attendance with severe frailty is as low as 70%.^18^ Timeframes on individual prognosis are fraught with uncertainty; however, the development of severe frailty is an indication that someone is reaching the last phase of their life. This is an opportunity to prepare and support patients and their families, including recognising and explaining ordinary dying.

Anticipatory care planning is important here. To have value, this needs to be specific. Families need to know what to do in a crisis when their loved one may not be able to communicate. Having clear assessment of the degree of frailty allows a structured approach to both prognosis and a clue to otherwise unexpected changes in health status, as well as a record of how rapidly things are changing and when plans may need to be revisited. Importantly, effective anticipatory care planning is rarely a one-off event, and people living with frailty typically need time and support to reflect and meaningfully reach a shared decision about care.^19^ Enabling such conversations to take place in the context of trust built over time is a central argument for prioritising continuity of care within general practice.

### More than a label

There is a tension that, in general, people rarely identify with the term frailty. It often carries negative connotations and people may fear being ‘written off’.^20^ These are important concerns. However, some of the greatest tragedies within our healthcare systems occur when frailty is not recognised. We can do great and inadvertent harm to people living with frailty through pathways and guidelines that are not attuned to their needs. Frailty assessment offers an opportunity to help patients (and carers) with care plans that allow individualised goals to be set. These identify targets for intervention to preserve or maximise independence, adjust approaches to managing individual conditions in ways that align with patients’ priorities and reduce avoidable harm, and allow plans to be made before point of crisis when there is time to establish trust and to find out what truly matters to people. One size does not fit all, and being intentional about patients’ degrees of frailty can allow us to deliver care that fits.
